# Contemporary Management of Hepatic Cyst Disease: Techniques and Outcomes at a Tertiary Hepatobiliary Center

**DOI:** 10.1007/s11605-020-04821-1

**Published:** 2020-10-20

**Authors:** Axel Gomez, Andrew D. Wisneski, Hubert Y. Luu, Kenzo Hirose, John P. Roberts, Ryutaro Hirose, Christopher E. Freise, Eric K. Nakakura, Carlos U. Corvera

**Affiliations:** Department of Surgery, University of California San Francisco, 533 Parnassus Avenue, Room 370, San Francisco, CA USA

**Keywords:** Liver cysts, Minimally invasive, Laparoscopy, Fenestration, Clinical outcomes

## Abstract

**Background:**

Hepatic cyst disease is often asymptomatic, but treatment is warranted if patients experience symptoms. We describe our management approach to these patients and review the technical nuances of the laparoscopic approach.

**Methods:**

Medical records were reviewed for operative management of hepatic cysts from 2012 to 2019 at a single, tertiary academic medical center.

**Results:**

Fifty-three patients (39 female) met the inclusion criteria with median age at presentation of 65 years. Fifty cases (94.3%) were performed laparoscopically. Fourteen patients carried diagnosis of polycystic liver disease. Dominant cyst diameter was median 129 mm and located within the right lobe (30), left lobe (17), caudate (2), or was bilobar (4). Pre-operative concern for biliary cystadenoma/cystadenocarcinoma existed for 7 patients. Operative techniques included fenestration (40), fenestration with decapitation (7), decapitation alone (3), and excision (2). Partial hepatectomy was performed in conjunction with fenestration/decapitation for 15 cases: right sided (7), left sided (7), and central (1). One formal left hepatectomy was performed in a polycystic liver disease patient. Final pathology yielded simple cyst (52) and one biliary cystadenoma. Post-operative complications included bile leak (2), perihepatic fluid collection (1), pleural effusion (1), and ascites (1). At median 7.1-month follow-up, complete resolution of symptoms occurred for 34/49 patients (69.4%) who had symptoms preoperatively. Reintervention for cyst recurrence occurred for 5 cases (9.4%).

**Conclusions:**

Outcomes for hepatic cyst disease are described with predominantly laparoscopic approach, approach with minimal morbidity, and excellent clinical results.

## Introduction

Hepatic cysts are found in 2.5–18% of the general population and carry a broad differential diagnosis^1^,^2^. Infectious etiologies include pyogenic abscess, amoebic abscess, and hydatid cyst, while non-infectious cysts include benign lesions such as simple liver cysts, ciliated foregut cysts, and Caroli disease^3^. Cystic neoplasms of the liver include entities such as biliary cystadenoma, biliary cystadenocarcinoma, and liver metastases. Non-infectious hepatic cyst disease is often an incidental finding on abdominal imaging or during abdominal surgery performed for other reasons. In spite of the fact that hepatic cysts are fairly prevalent, only 5–10% of patients become symptomatic^4^. Symptoms of non-infectious hepatic cyst disease can arise insidiously and may progress to debilitating abdominal pain, nausea, vomiting, and early satiety. Female gender and advanced age are the predominant risk factors for symptomatic hepatic cyst disease^4^. Less frequently, patients can present with a more acute course due to cyst hemorrhage, rupture, or infection^5^.

Simple liver cysts (SLCs) constitute the most common hepatic cyst diagnosis, and conservative management with observation is acceptable in the absence of symptoms or concerning radiographic features. Symptomatic SLC warrants intervention if substantial impact to quality of life develops. Several kinds of interventions can be offered to patients, ranging from less to more invasive. Non-surgical treatments include percutaneous aspiration and sclerotherapy, but their utility is plagued by high cyst recurrence rates^6^,^7^. Surgery for SLC offers patients a more definitive treatment, and a variety of operative interventions can be performed including cyst fenestration, cyst fenestration with decapitation, partial or formal hepatectomy, and rarely liver transplantation. Historically, operations to fenestrate or remove cysts were done as open procedures. With the advent of laparoscopic surgery and wide recognition of its benefits since the 1990s, certain patients with symptomatic SLC disease were ideal candidates for this approach^8^. This is because the anticipated specimen size is often small, and the majority of the cyst’s fluid is easily evacuated by suctioning. Nowadays, the open approach is traditionally reserved for diffuse disease, difficult cyst location (e.g., posterior or superior liver surface), or if neoplastic entity is highly suspected based on pre-operative imaging. With improvements in modern imaging, instrumentation, and surgical techniques, the optimal cyst intervention continues to evolve. Minimally invasive hepatobiliary surgery is now proven to be safe and effective for a variety of benign and oncologic diseases^9^,^10^. As such, our institution routinely performs laparoscopic liver surgery for minor and major hepatectomies. The aim of this study is to describe our experience and outcomes of non-infectious hepatic cyst disease management at a tertiary hepatobiliary center where the laparoscopic approach is standard.

## Materials and Methods

We conducted a review of medical records for patients at our institution that underwent surgical treatment for non-infectious hepatic cyst disease between June 2012 and August 2019. This study was approved by the Committee on Human Research at the University of California San Francisco Medical Center. Information on demographics, clinical presentation, baseline liver function tests, radiographic imaging, surgical intervention, and final specimen pathology was collected. Cases of liver transplantation for hepatic cyst disease were excluded. All patients had a pre-operative abdominal computed tomography (CT) scan. Magnetic resonance imaging (MRI) was obtained if the CT images demonstrated features suggestive of biliary cystadenoma or cystadenocarcinoma, such as cyst multi-loculation or septation, calcifications, nodules, or wall enhancement^11^. We also collected information on the post-operative course, symptom changes, and radiographic recurrences.

Continuous variables are reported as medians with interquartile ranges, while categorical variables are reported as counts and percentages. Variables were classified by both initial surgical approach and procedure. Comparison of categorical variables was performed with Fisher’s exact test; comparison of continuous variables was performed with the Mann-Whitney *U* test. A *p* value < 0.05 was considered statistically significant. Statistical analyses were performed using R (http://www.r-project.org).

## Results

### Demographics

A total of 53 consecutive patients underwent surgical intervention during the study period with a median age of 65 years. Fourteen (26.4%) patients carried a diagnosis of polycystic liver disease (PCLD). The majority of patients were American Society of Anesthesiology (ASA) class II, with hypertension and gastroesophageal reflux disease being the most prevalent co-morbidities. See Table 1 for complete demographic information.

**Table 1:** Patient characteristics

Characteristic	*N* (%) or median [IQR]
Total	53
Gender	
Male	14 (26.4%)
Female	39 (73.6%)
Age (years)	65 [60–71]
Body mass index (kg/m^2^)	23 [21.9–29.2]
ASA class	
1	4 (7.5%)
2	34 (64.2%)
3	15 (28.3%)
Pre-operative leading diagnosis	
Simple liver cyst	32 (60.4%)
Polycystic liver disease	14 (26.4%)
Suspected biliary cystadenoma or cystadenocarcinoma by imaging	7 (13.2%)
Co-morbidities	
Hypertension	23 (43.4%)
Gastroesophageal reflux disease	15 (28.3%)
Hyperlipidemia	14 (26.4%)
Asthma	5 (9.4%)
Benign prostatic hypertrophy	5 (9.4%)
Diabetes mellitus	5 (9.4%)
History of renal transplant	3 (5.7%)
Coronary artery disease	3 (5.7%)
Atrial fibrillation	2 (3.8%)
Systemic lupus erythematosus	1 (1.9%)

*ASA* American Society of Anesthesiology

### Symptoms and Previous Cyst Intervention

Nearly all patients reported symptoms that were attributable to liver cysts with median duration of approximately 1 year prior to the index intervention captured in this study. Ten patients (18.9%) had a previous intervention for SLC elsewhere, which included aspiration, fenestration, unroofing, and hepatectomy. Two patients had a prior left hepatectomy for symptomatic PCLD. The patients’ pre-operative symptoms and previous interventions for SLC are shown in Table 2.

**Table 2:** Symptoms, prior intervention, and baseline laboratory values

Characteristic	*N* (%) or median [IQR]
Symptoms	49 (92.5%)
Abdominal pain > 6 months duration	34 (64.2%)
Abdominal pain < 6 months duration	7 (13.2%)
Bloating	10 (18.9%)
Early satiety	10 (18.9%)
Shortness of breath	3 (5.7%)
Weight loss	2 (3.8%)
No symptoms	4 (7.5%)
Months from symptom onset to surgery	12 [4–12]
Prior liver cyst intervention	10 (18.9%)
Aspiration	5 (9.4%)
Left hepatectomy	2 (3.8%)
Unroofing	2 (3.8%)
Fenestration	1 (1.9%)
Pre-operative labs	
Creatinine (mg/dL)	0.80 [0.69–1.0]
Aspartate transaminase (U/L)	25 [21.5–33.0]
Alanine transaminase (U/L)	31.9 [16–36]
Total bilirubin (mg/dL)	0.7 [0.6–0.85]
Alkaline phosphatase (U/L)	89.3 [63.5–105.5]

### Pre-Operative Evaluation

Baseline liver function tests were within the reference ranges for 45 patients (85%), while mild derangements were found in the remaining eight patients. No patients had obstructive jaundice or required biliary decompression. The majority of patients had undergone liver ultrasound to initially diagnose liver cyst disease prior to referral to our institution. All patients considered for surgical intervention underwent pre-operative CT imaging, with liver MRI obtained if cysts demonstrated any concerning features suggestive of non-benign diagnosis. Seven patients’ cysts harbored radiographic features which prompted suspicion for a diagnosis of biliary cystadenoma or cystadenocarcinoma. The majority of patients had a single dominant cyst, with median cyst diameter of 129 mm, and right hepatic lobe predominance. Table 3 lists cyst characteristics.

**Table 3:** Operative characteristics

Characteristic	*N* (%) or median [IQR]
Number of dominant cysts present	
1	38 (71.7%)
2	14 (26.4%)
3	1 (1.9%)
Largest cyst diameter (mm)	129 [99–170]
Largest cyst estimated volume (cm^3^)	1123 [508–2572]
Predominant hepatic lobe involvement	
Left	17 (32.1%)
Right	30 (56.6%)
Bilobar	4 (7.5%)
Caudate	2 (3.8%)
Surgical approach	
Laparoscopic	50 (94.3%)
Open	2 (3.8%)
Laparoscopic converted to open	1 (1.9%)
Cyst intervention	
Fenestration	40 (75.5%)
Fenestration and decapitation	7 (13.2%)
Decapitation	3 (5.7%)
Excision	2 (3.8%)
Hepatectomy performed^a^	16 (30.2%)
Partial right hepatectomy^b^	7 (13.2%)
Partial left hepatectomy	7 (13.2%)
Formal left hepatectomy^b^	1 (1.9%)
Central hepatectomy	1 (1.9%)
Laparoscopic partial hepatectomy	14 (26.4%)
Signs of prior cyst hemorrhage found	18 (34.0%)
Concurrent cholecystectomy performed	16 (30.2%)
Omentalplasty to cyst cavity	10 (18.9%)

^a^All hepatectomies, except formal left hepatectomy case, performed in conjunction with fenestration, decapitation or excision

^b^One case of each performed as open approach

### Operative Characteristics

**Fig. 1:**
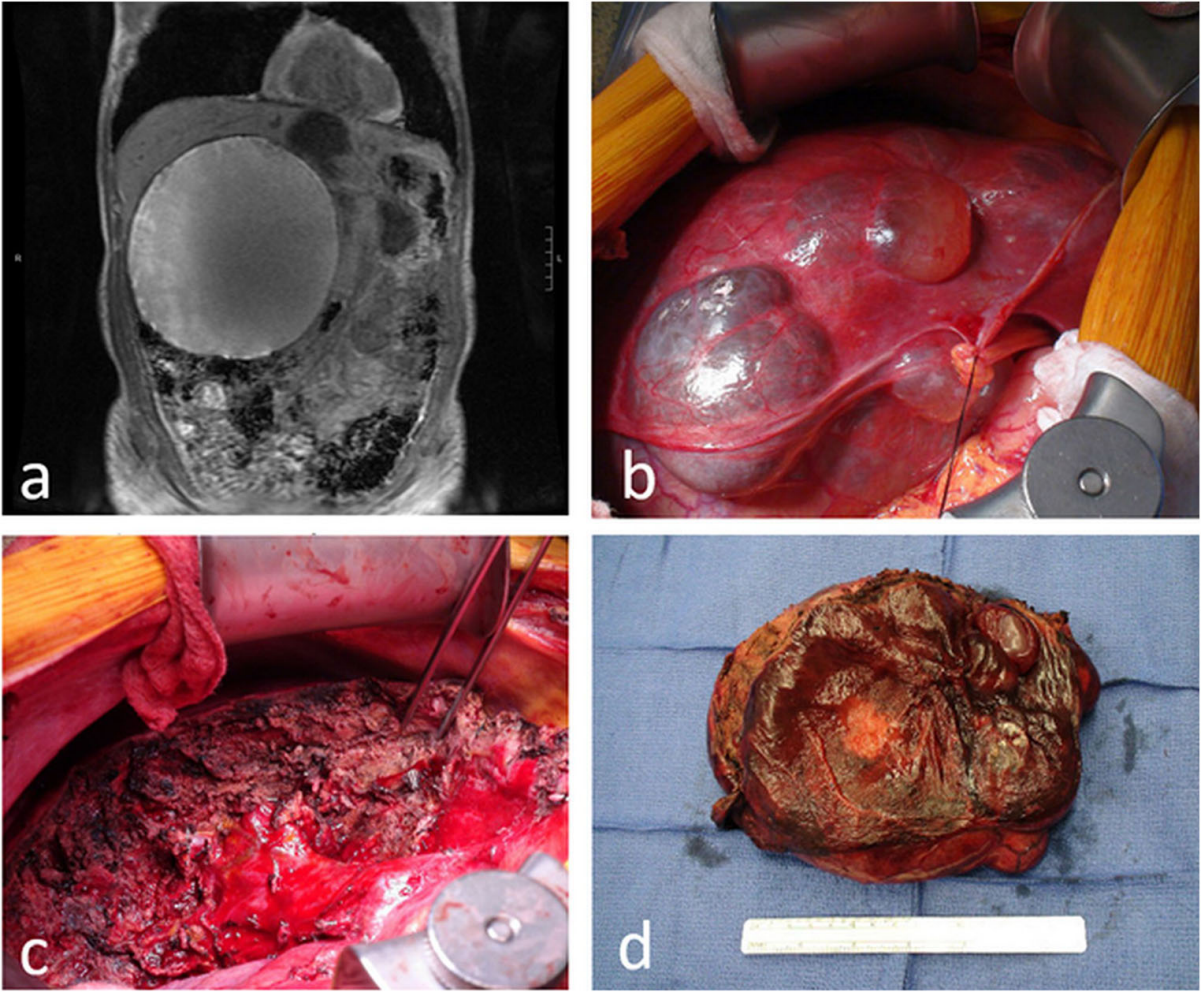
**a** Coronal magnetic resonance image of a 19-cm cyst in a 70-year-old man with features concerning for mucinous cystadenoma. **b** Anterior surface of the liver during open approach. **c** Transection line for partial segment IVa and IVb hepatectomy. **d** The cyst removed intact, which resulted with benign pathology

The vast majority of procedures (94.3%) were successfully performed with a completely laparoscopic approach. One laparoscopic procedure was converted to open in a PCLD patient who could not tolerate the insufflation. Primary laparotomy was planned in two cases (3.8%) due to high pre-operative suspicion for neoplastic disease (Fig. 1). Intra-operative frozen sections helped confirm the pathology and guide resection for these two cases. Cyst enucleation with clear margins was done for a segment IVb biliary cystadenoma, while the other returned as a large simple cyst and a partial left hepatectomy was carried out. Regarding cyst intervention technique, the majority of procedures (67.9%) were fenestrations with or without decapitation and without hepatectomy. Fifteen (28.3%) partial hepatectomies were performed, and one formal left hepatectomy was performed. Omentalplasty to the dominant cyst cavity was done in ten patients (18.9%), and concurrent cholecystectomy was performed in 16 patients (30.2%) to facilitate exposure to segments IVb or V, or to eliminate gallstones as a possible contributor to symptoms. Refer to Table 3 for operative details.

### Histopathology

The majority of the cysts were benign SLCs (98.1%), with two SLCs having associated focal nodular hyperplasia. One biliary cystadenoma was confirmed on pathology out of the seven cases where possible cystadenoma or cystadenocarcinoma was suspected based on pre-operative imaging. See Table 4 for final post-operative pathology results.

**Table 4:** Post-operative liver cyst histopathology

Characteristic	*N* (%)
Simple cyst	50 (94.3%)
Simple cyst with focal nodular hyperplasia	2 (3.8%)
Biliary cystadenoma	1 (1.9%)
Biliary cystadenocarcinoma	0 (0%)

### Post-Operative Care and Follow-Up

Post-operative complications occurred in 20.8% of patients (11/53). One patient, who underwent laparoscopic converted to open formal left hepatectomy, required transfusion of two units of red blood cells in the immediate post-operative period for bleeding, but had no further issues. One patient developed a small right-sided pleural effusion managed with diuretics and required no further intervention. Four Clavien-Dindo class IIIa complications occurred and constituted the highest-grade events in this cohort^12^. Two patients (3.8%), one with PCLD, developed post-operative bile leaks that were successfully managed by endoscopic retrograde cholangiopancreatography (ERCP) with biliary stenting. One PCLD patient developed new-onset post-operative ascites requiring paracentesis that resolved several weeks after discharge. Another patient developed a sterile peri-hepatic fluid collection that was treated with percutaneous drainage. The overall complication rate of PCLD patients was greater than that of non-PCLD patients, but this did not reach statistical significance (21.4% vs 7.7%, *p* = 0.16). The median length of hospital stay was 2 days, and the 30-day post-operative readmission rate was 3.8% (two patients); one for management of a perihepatic fluid collection by percutaneous drainage, and one patient who presented with nausea, emesis, and fevers which was attributed to a viral illness as no intra-abdominal pathology was identified. Post-operative mortality within 90 days was 0.0%.

The median post-operative follow-up duration was 7.1 months. Of the 49 patients who had symptoms attributed to their hepatic cysts pre-operatively, 69.4% reported complete resolution of symptoms, 24.5% experienced partial resolution, and 6.1% remained with ongoing symptoms. Our cyst recurrence rate, which we define as radiographic evidence of cyst recurrence, was 22.6% (12/53). Recurrent cysts were always smaller than the originally treated ones. Median time to cyst recurrence diagnosis was 18.2 months after surgery. Radiographic recurrence rates in patients with and without PCLD were 35.7% versus 17.9% (*p* = 0.17). Recurrence rates in patients who underwent hepatectomy versus no hepatectomy were 31.3% versus 18.6% (*p* = 0.48). Six of the 12 recurrences had cyst involvement of segments VI, VII, and/or VIII. Five cases of recurrence were symptomatic resulting in patients undergoing additional intervention a median 36.5 months after the index intervention. Three patients had repeat fenestration while two received percutaneous aspiration. A summary of follow-up evaluation is reported in Table 5.

**Table 5:** Post-operative course and follow-up

Characteristic	*N* (%) or median [IQR]
Hospital length of stay (days)	2 [1–3]
Any complication occurrence	11 (20.8%)
Clavien-Dindo grade complication occurrence	
I	5 (9.4%)
II	2 (3.8%)
IIIa	4 (7.5%)
Post-operative complications requiring intervention	
Biliary leak	2 (3.8%)
New ascites	1 (1.9%)
Perihepatic fluid collection	1 (1.9%)
Bleeding requiring transfusion	1 (1.9%)
30-day readmission	2 (3.8%)
Symptom resolution at follow-up	
Complete	34/49 (69.4%)
Partial	12/49 (24.5%)
Ongoing symptoms	3/49 (6.1%)
Follow-up duration (months)	7.1 [0.9–22.9]
Cyst recurrence on imaging	12 (22.6%)
Months to recurrence diagnosis	18.2 [12.7–53.5]
Cyst recurrence requiring repeat intervention	5 (9.4%)
Repeat fenestration	3 (5.7%)
Percutaneous aspiration	2 (3.8%)

## Discussion

This study describes non-infectious liver cyst disease management at a tertiary hepatobiliary surgical center. We performed 94.3% of surgical procedures with a totally laparoscopic approach which constitutes one of the highest reported rates for a single institution’s laparoscopic approach to simple hepatic cyst disease. Other studies report laparoscopic approaches for hepatic cyst disease in the range of 60–80% range^13^–^15^. Aside from patient co-morbidities, a cyst having atypical features, being deeply situated in the parenchyma, or being in a difficult anatomic location have been traditional factors limiting use of the laparoscopic approach^14^,^15^. In our cohort, 33.9% of cases had dominant cyst involving liver segments VI, VII, or VIII, which have been considered relatively more challenging to access by laparoscopy^15^,^16^. Our results demonstrate that cyst location is not a contraindication to a safe and effective laparoscopic approach. Our surgical group possesses expertise in minimally invasive liver surgical techniques, thus access to and resection of any portion of the liver does not pose a contraindication to a totally laparoscopic approach.

Regarding our patient selection for surgical treatment of SLC, not all symptomatic patients are offered immediate surgical intervention. Since the natural history of SLC predicts slow growth over the years, it is acceptable to follow some patients with short interval imaging. An exception is the small percentage of patients with SLC that can experience a sudden increase in cyst size related to internal hemorrhage. We recommend ultrasound or axial imaging in patients with small cysts < 5 cm in awkwardly positioned areas within the liver such as those situated in the caudate process displacing the portal structures anteriorly and the inferior vena cava posteriorly. These cysts often require a partial hepatectomy to gain access to the cyst wall, and they are difficult to fenestrate widely to prevent recurrence. Another cyst position that is challenging to manage is the right superior, posterior location. In young healthy non-symptomatic patients with cysts in this location of moderate size ~ 10 cm, we recommend close surveillance and intervene for development of symptoms, or rapid interval rate of growth (i.e., > 3 cm/year). Although controversial, we recommend surgical fenestration in non-symptomatic patients with large > 15 cm cysts in this location especially older healthy patients. This recommendation comes from our experience in managing several patients with cyst recurrence in this location from failed laparoscopic and open procedures. The rational for early intervention is to prevent irreversible diaphragmatic eventration, and insidious pulmonary compromise.

Patient co-morbidity was the major factor for our sole case of unplanned laparotomy. Our study’s population was relatively healthy with 72% of patients being ASA class I or II. The only conversion from laparoscopic to open approach occurred in an ASA class III patient with underlying pulmonary disease who was unable to tolerate the laparoscopic insufflation. No issues pertaining to technical difficulty of the cyst intervention, hepatic injury, or exposure necessitated the conversion. Planned primary laparotomy was performed for two cases where pre-operative suspicion for biliary cystadenoma was high. Pre-operative suspicion for non-benign diagnosis coupled with surgeon experience should guide the overall operative approach.

Simple cysts accounted for 98% of the cysts encountered in our patients. In most series on liver cyst management, neoplastic lesions represent < 5% of the pathologic diagnoses which is consistent with our experience^7^,^17^. Cross-sectional imaging, while effective at characterizing simple cysts, is limited in providing highly accurate differentiation of more complex cystic lesions 11,18. In absence of pronounced lab abnormalities, intra-hepatic biliary duct dilatation, and numerous hallmark features within the cyst, there is a potential for a fair amount of diagnostic uncertainty until the specimen is resected and undergoes histopathologic analysis. Illustrative of this uncertainty is the fact that out of seven cases with pre-operative suspicion for neoplasm, one returned as neoplasm on pathology. This was also one of two cases where suspicion was strong enough as to justify a primary open approach. These two particular cases included a 70-year-old man with a 19-cm cyst with the pre-operative MRI highly suggestive of mucinous cystadenoma and a 74-year-old woman with a cyst that had grown from 2.0 to 5.5 cm over 2 years with formation of solid internal components and signs of intra-cystic hemorrhage. The former case returned as benign simple cyst with fibrosis and hemorrhage, while the latter returned as a biliary cystadenoma. Thus, even simple cysts can mimic neoplastic lesions especially with internal cyst hemorrhage.

We advocate for an oncologically sound approach to the management of these lesions if pre-operative evaluation was to suggest possibility of biliary cystadenoma or cystadenocarcinoma. Importantly, the preoperative informed consent discussion with the patient should detail the possibility of conversion to an open procedure with more extensive resection if cancer were to be confirmed intra-operatively. We recommend use of intra-operative frozen section analysis as an aide to the surgical plan and frequently used this to evaluate nodules or other findings on cases even when benign SLC was the leading pre-operative diagnosis. We advise careful and thorough inspection of the cyst wall intra-operatively. After cyst fenestration and fluid evacuation, the entire cyst wall should be carefully inspected for any suspicious mural nodules. Hemorrhagic cysts with resorbed clot and debris can obscure features of the cyst wall and should be gently irrigated and suctioned. Removing as much of the cyst wall not in direct contact with the liver parenchyma aides in more throughout inspection after removal. Any suspicious areas should be marked by the surgeon to help orient the specimen for frozen section analysis. The surgeon should also be prepared to change the operative plan accordingly if a neoplastic diagnosis resulted from pathology. Whether it be to continue laparoscopically, or convert to an open approach, all potential diagnoses and surgical plans need to be considered to perform the appropriate treatment.

Our results demonstrate that predominant use of laparoscopic cyst treatment extends the benefits of minimally invasive surgery to patients with reduced recovery times, shorter in-hospital stays, and morbidity comparable with general literature rates^19^–^21^. Aside from the one patient who was unable to tolerate insufflation, there were no intra-operative adverse events as a result of technical factors from the cyst surgery. Studies cite intra-operative adverse events up to 15% with inadvertent liver parenchymal injury being the most common occurrence 15, 22. Our overall post-operative complication rate was 20.8%. All Clavien-Dindo grade III complications resolved without issue. Our rate of bile leak (3.8%, *n* = 2) and post-operative ascites (1.9%, *n* = 1) is commensurate to other published studies whose rates are generally under 6%^13^,^15^,^23^–^28^.

There was a higher overall complication rate in patients with PCLD. This finding is consistent with heightened morbidity reported surrounding cyst treatment in PCLD patients, given more extensive disease burden and the increased technical challenges with resection in these patients^23^,^29^,^30^. However, this was not statistically significant compared with the overall complication rate in non-PCLD patients, and our study was not specifically powered to examine this outcome.

In this study, 23% of patients had radiographically proven cyst recurrence during the median 7 months follow-up period. Re-intervention for symptomatic recurrence occurred in 9.4% of the study population. Our results are consistent with studies that define recurrence based on imaging. A study that incorporated routine sonographic evaluation on follow-up reported a recurrence rate of 28%^15^. Similar to our approach, when radiographic evaluation was dictated by persistent or worsening symptoms, studies found rates of 12–20%^22^, ^31^. There was only one radiographic recurrence in patients who underwent hepatectomy. Partial hepatectomy is a more extensive procedure but has been shown to be highly effective in the management of SLCs with extremely low rates of recurrences, oftentimes under 1%^14^, ^22^, ^32^. Although a more demanding procedure, hepatectomy can help avoid cyst recurrence by producing improved drainage of any retained cyst wall directly into the peritoneal cavity for resorption. This avoids cyst wall reformation that can occur if the overlying diaphragm collapses over the hepatic defect leading to fluid accumulation, even if omentalplasty has been done to the cyst cavity. Regardless of the approach, overall reintervention rates due to cyst recurrence are in the range of 5–10% across studies^15^,^22^,^31^,^33^.

Thirty percent of patients experienced only partial resolution or reported the same symptom severity after the cyst treatment. Similarly, studies that obtained follow-up beyond 2 years reported 20–50% rates of persistent symptoms after intervention^33^–^35^. Interestingly, Scheuerlein and colleagues found nearly half of patients who did not experience complete resolution of symptoms reported an increase in symptom severity^15^. There is a discrepancy in the number of patients that continue to experience symptoms and those proven to have recurrent cyst disease on imaging. This observation may be explained by the non-specific nature of liver cyst symptoms which may overlap with those from other etiologies (i.e., gallstones, musculoskeletal, functional gastrointestinal disorders). As such, surgical management with concomitant cholecystectomy at time of cyst intervention allows elimination of gallstone disease as a source of persistent symptoms^23^.

Omentalplasty to the dominant cyst cavity was performed in 19% of cases, and of these, nearly one third experienced a radiographic recurrence. The effectiveness of omentalplasty at reducing cyst recurrence remains to be elucidated^23^,^24^,^36^. The intra-operative decision to perform omentalplasty was based on favorable cyst anatomy and whether the patient’s omentum could easily reach the dominant cyst cavity without additional extensive dissection. Overall, our outcomes remain comparable with the literature reported rates for morbidity and cyst recurrence, while offering one of the highest rates of a totally laparoscopic intervention.

This study is limited by its retrospective nature. Additionally, due to a predominant use of laparoscopy as the standard approach for most of our patients, it is difficult to effectively compare outcomes with the open approach or with percutaneous drainage. Another limitation is a relatively short post-operative follow-up duration of median 7 months. As a result of referral patterns, many patients reside long distances from our medical center and return to local care after the first post-operative visit if no issues are identified. For 17 patients, the immediate post-operative clinic visit within 30 days of procedure served as the only form of follow-up. Furthermore, post-operative imaging was not obtained unless persistent clinical symptoms prompted further evaluation for recurrent cyst or other pathology. Thus, the asymptomatic cyst recurrence rate is difficult to ascertain. This is further confounded by the possibility of a smaller cyst growing or de novo cyst formation counting as a recurrence from incomplete treatment versus new disease. A longitudinal study with standard post-operative imaging surveillance and longer-term clinical follow-up would be needed to capture true cyst recurrence rates.

## Conclusion

This study describes management of hepatic cyst disease at a tertiary academic hepatobiliary center and is notable for having one of the highest literature-reported rates of a totally laparoscopic approach. With appropriate hepatobiliary surgical expertise in minimally invasive techniques, difficult anatomic locations should not preclude a laparoscopic approach nor achievement of excellent clinical outcomes with low morbidity. If cystic neoplasm is suspected, use of intra-operative frozen section is helpful to guide resection, and the surgeon should be prepared to perform the appropriate oncologic resection.
